# Editorial: Plant microbiome: Diversity, functions, and applications

**DOI:** 10.3389/fmicb.2022.1039212

**Published:** 2022-09-26

**Authors:** Khondoker M. G. Dastogeer, Jenny Kao-Kniffin, Shin Okazaki

**Affiliations:** ^1^Department of Plant Pathology, Bangladesh Agricultural University, Mymensingh, Bangladesh; ^2^Department of Environmental Science, Policy, and Management, University of California, Berkeley, Berkeley, CA, United States; ^3^Horticulture Section, School of Integrative Plant Science, Cornell University, Ithaca, NY, United States; ^4^Plant Microbiology Laboratory, Tokyo University of Agriculture and Technology, Tokyo, Japan

**Keywords:** endophytes, metabolites, microbiome diversity, plant health, plant microbiome

Considerable attention on plant microbiomes has been growing over the past decade featuring model plants, natural, and agricultural crop systems (Berg et al., 2020; Chialva et al., 2022). Studies are being carried out to understand microbial community composition, assembly process and underlying factors, evolution and ecology, and functional roles (Compant et al., 2019; Dastogeer et al., 2020; Xu et al., 2021; Trivedi et al., 2022). The power of advanced omics and multi-omics techniques, computational power, biostatistics, and remodeling of biological and evolutionary theories allows for a deeper understanding of plant—-microbiome interactions (Subramanian et al., 2020; Xu et al., 2021). The study of how the microbiome interacts with other elements of the environment is gaining momentum, and efforts are being made to understand the functional roles of the microbiome in translating microbiome potentials into sustainable plant production and protection. Currently, the engineering of microbial flora and the development and application of the synthetic community (SynComs) approach have revealed an essential role of microorganisms in plant adaptability and productivity (Liu et al., 2019; Nerva et al., 2022).

This Research Topic hosts eleven original research articles and a review article. The original research articles span experimental findings of various plants with their associated microbes, such as apple, aspen, avocado, chrysanthemum, eucalyptus, licorice, morning glories, tea, tobacco, and yellow witchweed.

## Crop management modulates microbiome diversity and composition

It is increasingly evident that crop management practices, including plant protection measures, influence the microbiome of plants and soil (Jing et al., 2022). The study by Wang et al. substantiates that monoculture practice significantly modulates microbiome compositions. They reported that continuous monocropping changed the profiles of the bacterial and fungal communities in cut chrysanthemum. The longer the monocropping time, the greater the reduction of biocontrol bacteria but the augmentation of pathogenic microbes in the rhizosphere of plants. These changes in microbial composition were also correlated with concomitant changes in soil properties in the monoculture practice.

*Glycyrrhiza uralensis* known as licorice is one of the most commonly used bulk medicinal materials in traditional Chinese medicine (Jiang et al., 2020). The quality of the desired product depends on many factors, including the techniques of licorice processing. Li et al. studied the association of endophytic microorganisms and the accumulation of metabolites in different licorice drying methods. Natural drying impacted the microbial diversity of licorice and Nectriaceae, with Enterobacteriaceae becoming more abundant under these conditions. They reported a correlation between dominant endophyte taxon and the metabolite types present in fresh vs. dry processing, suggesting that fresh-processing is an effective drying method to ensure the quality of licorice.

## Association of the microbiome with plant health status

Understanding the role of the microbiome in plant health is essential in pinpointing the etiology of disease and devising appropriate management solutions (Dastogeer et al., 2022; Trivedi et al., 2022). Mahnkopp-Dirks et al. conducted experiments with apple replant disease (ARD) to understand whether it influences structuring plant microbiome composition and diversity. This disease has worldwide occurrence in many tree nurseries, but there still remains a lack of knowledge about the cause and management of this complex disease (Mazzola and Manici, 2012; Winkelmann et al., 2019). Mahnkopp-Dirks et al. used culture-independent and culture-dependent methods to compare the endophytic root microbiome of apple plants grown in ARD-affected soil vs. those in control soil. They reported that several *Streptomyces* increased over time, particularly in virgin soil, and based on this, they speculated that Streptomyces might be associated with ARD etiology. Also, ARD-affected soil had a reduced abundance of *Pseudomonas* roots endophytes, indicating its importance for a balanced microbiome in healthy soils. Culturing bacteria in media also showed a high diversity of *Pseudomonas*, supporting the view of their microbiome study. Ahmed et al. employed similar methods to gain a microbiome perspective of bacterial wilt-infected Flue-Cured Tobacco (*Nicotiana tabacum*) plants. They reported that the healthy plants exhibited higher microbial diversity than diseased plants with increased abundances of several bacteria such as *Bacillus, Bradyrhizobium, Ensifer, Neorhizobium*, and *Lysobacter*, which have been variously reported to be plant growth promoters and disease suppressor. There were increased abundances of bacterial wilt pathogen *Ralstonia solanacearum* rhizosphere soil and root of diseased plants. In addition, several fungi commonly identified as plant pathogens, *Plectosphaerella cucumerina, Alternaria alternata, Thanatephorus cucumeris*, and *Fusarium* sp., were encountered more frequently in the rhizosphere soil and root. They hypothesized that higher abundances of beneficial bacteria in the healthy plants and pathogenic fungi in diseased plants may be associated with the bacterial wilt disease in tobacco and that the complexity of bacterial and fungal communities possibly interact with each other (microbe–microbe) and host (host-microbe) to influence growth and induce resistance against bacterial wilt disease.

Cao et al. showed that infection of root-knot nematode (RKN) causes significant alteration in the diversity and composition of root microorganisms in tobacco. While the diversity of rhizosphere bacteria was reduced, the diversity of rhizosphere fungi increased upon infection by RNK. In particular, the abundance of *Rhizobiaceae* and *Chryseobacterium* increased after RKN infection. Nematode infection altered the overall composition of root microbiome but only at specific growth stages of plants, indicating variable nematode-microbe-plant interactions at different development stages of plants. Farooq et al. conducted microbial profiling of rhizosphere soil and root endosphere of avocado plants infested or not infested with *Phytophthora cinnamomi*. They compared the changes in microbial diversity and community composition of infested and non-infested plants grown with various soil additives or by spraying plants with phosphite. The results revealed phosphite treatment or soil application of mineral mulch applied to the soil reduced *Phytophthora* infestation. Bacterial abundance and diversity were reduced in infested rhizospheres and root endospheres. The mineral mulch application significantly changed diversity and rhizosphere community composition more than any other treatment. Some rhizosphere bacterial groups, especially Actinobacteria and Proteobacteria, had significantly higher relative abundance in the presence of *Phytophthora*. The bacterial communities of root endospheres were lower in abundance than rhizosphere communities and not affected by soil treatments or phosphite but increased in abundance after infection with *P. cinnamomi*. These findings suggested that adding silicate-based mineral mulch protects against *Phytophthora* root rot, which changes in rhizosphere bacterial community composition may partly mediate. However, the changes to the microbiome induced by spraying plants with phosphite are different from those resulting from applying mineral mulch to the soil. Plant fungal communities are diverse and influenced by many biotic and abiotic factors. The influence of biotic factors, however, is still poorly understood. Messal et al. investigated how insect infestation and gall formation on *Eucalyptus* foliage influence the diverse community composition of foliar fungi in *Eucalyptus grandis* trees. The infestation of the wasp *Leptocybe invasa* (Eulophidae: Hymenoptera) indeed brought about significant changes in the composition and diversity of fungal communities and was correlated with the severity of infestations. Their network analysis indicated that the co-occurrence of potential pathogens differed significantly between no to mild and medium to heavy infestation. This study increased our microbial interactions, especially the role of pathogens which may help design management approaches for pests employing beneficial host-associated microbial communities.

## Endophytes: Diversity, composition, evolution, and sources of novel metabolites

All plants in natural ecosystems appear to be symbiotic with fungal and bacterial endophytes, and their diversity is very high (Rodriguez et al., 2009; Afzal et al., 2019). They play important roles in helping plant growth and development. Tea is an economically important crop worldwide. The information about the distribution pattern and potential functions of endophytic communities in tea trees has been limited. Lin et al. investigated the diversity and community composition of endophytic bacteria and fungi of tea plants using amplicon sequencing. Ecological niches shape the diversity of endophytic microorganisms. They found that bacterial diversity was highest in root tissues followed by the stem, old leaves, and new leaves, and fungal diversity was highest in the old leaves followed by new leaves, roots, and stems. The cooperative relationship between endophytic bacteria and fungi in the new leaves was more substantial than that in the old leaves, which can better participate in the metabolism of tea material. Microorganisms, specifically the endophytes of medicinal plants, are recognized as valuable sources of novel secondary metabolites. Maela et al. isolated and identified five bacterial endophytes, *Alectra sessiliflora*, an important medicinal plant. *Bacillus* was the most dominant bacterial endophyte with two other genera*: Lysinibacillus* and *Peribacillus*. The crude extracts of three bacterial strains showed more than 90% growth inhibition against all the cancer cell lines at a concentration of 1,000 μg/ml. They also performed untargeted secondary metabolite profiling and reported the presence of compounds known to have biological activity. In addition to various abiotic and biotic factors, plant-fungal communities are also shaped by management factors. Witzell et al. hypothesized that nitrogen fertilization (N) modulates the quality of aspen (*Populus tremula*) leaves and thus alters the internal “chemical landscape” for the fungi. The study suggested that nitrogen treatment reduced foliar concentrations of CT precursor catechin but not that of condensed tannins (CTs). The N application also boosted the level of the amino acids and lowered the level of certain sugars. When a beetle herbivore was used as a treatment, no significant alteration in chemical composition or endophyte diversity was noted. A few rare fungi were associated with and potentially vectored by the beetle herbivores. They inferred that under controlled conditions and in a short period, the growth conditions, not the internal chemical quality, are the moderators of the fungal diversity in aspen leaves. The production of bioactive metabolites by endosymbiotic fungi is particularly important to the fitness of the fungus and the host species. The benefits and costs of symbiosis are not well-understood in many cases. The fungal endosymbiont of several plants, locoweeds (Fabaceae), morning glories (Convolvulaceae), and two species of Malvaceae are known to produce swainsonine. Swainsonine has toxic effects on livestock that feed on these plants and has potential pharmaceutical applications. Quach et al. evaluated 244 morning glory species for the presence of swainsonine and built a phylogeny based on available internal transcribed spacer (ITS) sequences. Only a single morning glory clade has swainsonine, and this symbiosis developed ~5 million years. Various orders of swainsonine-producing fungal endosymbionts in different plant families are genetically diverse. Further research would be exciting to know whether different symbioses have similar effects on host plants from other families and fungal-host specificity.

Gruet et al. provide an excellent review that sheds light on our current understanding of the microbiome of wheat root and rhizosphere based on pre-and post-domestication wheat history. Wheat, one of the major crops in the world, has had a complex history that includes genomic hybridizations between *Triticum* and *Aegilops* species and several domestication events. The author discusses differences between wild and domesticated wheat, ancient and modern types of cultivars, as well as individual cultivars within a given wheat species. Their analyses pointed out two significant trends. The majority of the investigations were carried out toward taxonomic diversity rather than the microbial functioning microbiota and more for bacteria and mycorrhizal fungi. Second, studies primarily compared wheat genotypes of *T. aestivum* cultivars, sometimes with little consideration for their particular genetic and physiological traits. The author hoped that the employment of current state-of-the-art sequencing technologies would enable revisiting the wheat microbiome's diversity to understand the wheat evolutionary history's significance better. This would provide the baseline information needed to develop microbiome-based breeding strategies for sustainable wheat farming.

The twelve articles comprising this special topic on *Plant Microbiome: Diversity, Functions, and Applications* highlight the advances and progress in research on a great diversity of plant species globally. The rapid developments in omics technologies and bioinformatics analysis are continuing to evolve the microbiome science field forward. We can expect many more novel discoveries in the coming years featuring innovative experimentation and predictive analyses.

## Author contributions

KD: conceptualization and draft preparation. JK-K and SO: revision and modification. All authors contributed to the article and approved the submitted version.

## Conflict of interest

The authors declare that the research was conducted in the absence of any commercial or financial relationships that could be construed as a potential conflict of interest.

## Publisher's note

All claims expressed in this article are solely those of the authors and do not necessarily represent those of their affiliated organizations, or those of the publisher, the editors and the reviewers. Any product that may be evaluated in this article, or claim that may be made by its manufacturer, is not guaranteed or endorsed by the publisher.
